# Recombinant human T-cell leukemia virus types 1 and 2 Tax proteins induce high levels of CC-chemokines and downregulate CCR5 in human peripheral blood mononuclear cells

**DOI:** 10.1186/1742-4690-8-S1-A98

**Published:** 2011-06-06

**Authors:** Christy S Barrios, Muna Abuerreish, Laura Castillo, Michael D Lairmore, Edward L Murphy, Chou-Zen Giam, Mark A Beilke

**Affiliations:** 1 Division of Infectious Diseases, Medical College of Wisconsin and Research Service, Clement J Zablocki Veterans Affairs Medical Center, Milwaukee, WI 53226, USA; 2 Department of Veterinary Biosciences, Center for Retrovirus Research, The Ohio State University, Columbus, OH, 43210, USA; 3 Departments of Laboratory Medicine and Epidemiology/Biostatistics, University of California, San Francisco, CA, 94118, USA; 4 Department of Microbiology and Immunology, Uniformed Services University of the Health Sciences, Bethesda, MD, 20814, USA

## Background

HTLV-1 and HTLV-2 trans-activating proteins (Tax1, Tax2) differ with respect to their ability to activate genes regulating viral replication, latency, and host cellular immune responses. Although HTLV-2 infections are usually asymptomatic, immunologic alterations are observed. We previously showed that HTLV-2 tax/rex viral load was upregulated in patients with HIV-1/HTLV-2 co-infection. This correlated with higher CD4+ T cell counts and improved health outcomes, possibly due to induction of CC-chemokines.

## Methods

In this study, recombinant Tax1 and Tax2 proteins were expressed in E. coli. PBMCs were incubated with different concentrations of Tax proteins (10-100 pM). Supernatant fluids and cells were harvested at multiple time points for quantitative determinations of MIP-1alpha/CCL3, MIP-1beta/CCL4, RANTES/CCL5, and CCR5 receptor expression.

## Results

In preliminary experiments it was shown that PMBCs from HTLV-2 infected donors had significantly lower levels of CCR5 expression and higher levels of RANTES/CCL5 compared to HTLV-2 seronegative donors (p<0.05). Tax1 and Tax2-treated PBMCs showed increased viability over a seven day period compared to controls (p<0.01). Both Tax1 and Tax2 induced equally high levels of all three CC-chemokines compared to mock-treated controls (p<0.05). Tax2-treated PBMCs showed a significantly lower percentage of CCR5-PE positive cells compared to mock-treated PBMCs within the gated lymphocyte population (p<0.05).

## Conclusions

Recombinant Tax2 is a potent modulator of CC-chemokines and CCR5 in vitro. Further investigations are needed to determine the underlying mechanism(s), and whether Tax2 recapitulates the observed effects in vivo.

